# Conventional MRI-Based Structural Disconnection and Morphometric Similarity Networks and Their Clinical Correlates in Multiple Sclerosis

**DOI:** 10.1212/WNL.0000000000213349

**Published:** 2025-01-23

**Authors:** Mario Tranfa, Maria Petracca, Marcello Moccia, Alessandra Scaravilli, Frederik Barkhof, Vincenzo Brescia Morra, Antonio Carotenuto, Sara Collorone, Andrea Elefante, Fabrizia Falco, Roberta Lanzillo, Luigi Lorenzini, Menno M. Schoonheim, Ahmed T. Toosy, Arturo Brunetti, Sirio Cocozza, Mario Quarantelli, Giuseppe Pontillo

**Affiliations:** 1 Department of Advanced Biomedical Sciences, University “Federico II,” Naples, Italy;; 2 Department of Radiology and Nuclear Medicine, MS Center Amsterdam, Amsterdam Neuroscience, Amsterdam UMC, Vrije Universiteit Amsterdam, the Netherlands;; 3 Department of Human Neurosciences, Sapienza University of Rome, Italy;; 4 Multiple Sclerosis Unit, Policlinico Federico II University Hospital, Naples, Italy;; 5 Department of Molecular Medicine and Medical Biotechnology, Federico II University of Naples, Italy;; 6 Centre for Medical Image Computing, University College London, United Kingdom;; 7 Dementia Research Centre, UCL Queen Square Institute of Neurology, University College London, United Kingdom;; 8 Queen Square Multiple Sclerosis Centre, Department of Neuroinflammation, UCL Queen Square Institute of Neurology, University College London, United Kingdom;; 9 Department of Neurosciences and Reproductive and Odontostomatological Sciences, University of Naples “Federico II,” Italy;; 10 Department of Anatomy and Neurosciences, MS Center Amsterdam, Amsterdam Neuroscience, Amsterdam UMC, Vrije Universiteit Amsterdam, the Netherlands; and; 11 Institute of Biostructure and Bioimaging, National Research Council, Naples, Italy.

## Abstract

**Background and Objectives:**

Although multiple sclerosis (MS) can be conceptualized as a network disorder, brain network analyses typically require advanced MRI sequences not commonly acquired in clinical practice. Using conventional MRI, we assessed cross-sectional and longitudinal structural disconnection and morphometric similarity networks in people with MS (pwMS), along with their relationship with clinical disability.

**Methods:**

In this longitudinal monocentric study, 3T structural MRI of pwMS and healthy controls (HC) was retrospectively analyzed. Physical and cognitive disabilities were assessed with the expanded disability status scale (EDSS) and the symbol digit modalities test (SDMT), respectively. Demyelinating lesions were automatically segmented, and the corresponding masks were used to assess pairwise structural disconnection between atlas-defined brain regions based on normative tractography data. Using the Morphometric Inverse Divergence method, we computed morphometric similarity between cortical regions based on FreeSurfer surface reconstruction. Using network-based statistics (NBS) and its extension NBS-predict, we tested whether subject-level connectomes were associated with disease status, progression, clinical disability, and long-term confirmed disability progression (CDP), independently from global lesion burden and atrophy.

**Results:**

We studied 461 pwMS (age = 37.2 ± 10.6 years, F/M = 324/137), corresponding to 1,235 visits (mean follow-up time = 1.9 ± 2.0 years, range = 0.1–13.3 years), and 55 HC (age = 42.4 ± 15.7 years; F/M = 25/30). Long-term clinical follow-up was available for 285 pwMS (mean follow-up time = 12.4 ± 2.8 years), 127 of whom (44.6%) exhibited CDP. At baseline, structural disconnection in pwMS was mostly centered around the thalami and cortical sensory and association hubs, while morphometric similarity was extensively disrupted (*p*_FWE_ < 0.01). EDSS was related to frontothalamic disconnection (*p*_FWE_ < 0.01) and disrupted morphometric similarity around the left perisylvian cortex (*p*_FWE_ = 0.02), while SDMT was associated with cortico-subcortical disconnection in the left hemisphere (*p*_FWE_ < 0.01). Longitudinally, both structural disconnection and morphometric similarity disruption significantly progressed (*p*_FWE_ = 0.04 and *p*_FWE_ < 0.01), correlating with EDSS increase (ρ = 0.07, *p* = 0.02 and ρ = 0.11, *p* < 0.001), while baseline disconnection predicted long-term CDP (accuracy = 59% [58–60], *p* = 0.03).

**Discussion:**

Structural disconnection and morphometric similarity networks, as assessed through conventional MRI, are sensitive to MS-related brain damage and its progression. They explain disease-related clinical disability and predict its long-term evolution independently from global lesion burden and atrophy, potentially adding to established MRI measures as network-based biomarkers of disease severity and progression.

## Introduction

Multiple sclerosis (MS) is a chronic neuroinflammatory and neurodegenerative disease of the CNS, commonly associated with physical disability and cognitive impairment, and carrying an important personal and socioeconomic burden.^1^ Although the assessment of focal lesions and brain atrophy using conventional MRI are crucial in the clinical management, they only partially explain the clinical heterogeneity observed in people with MS (pwMS).^2^

From the field of network neuroscience, conceptualizing the brain as a complex system of gray matter (GM) regions—nodes—linked by structural and functional connections—edges, MS can be modeled as a network disorder.^3,4^ Demyelinating lesions disrupt white matter (WM) pathways,^5^ while atrophy subverts the ordered patterns of morphometric similarity between GM areas.^6^ Throughout MS, the accumulation of structural damage affects the brain's functional organization, leading to physical disability and cognitive impairment.^4^ Shifting the emphasis from characterizing damage in specific regions to understanding network-level alterations has yielded unprecedented insights into the pathophysiologic mechanisms that underlie MS-related brain damage and associated clinical manifestations.^3^ However, brain network analyses are typically dependent on advanced MRI sequences, hampering their implementation in clinical settings. This has led to considerable efforts in developing network analyses using anatomical images to enable the (re)analysis of conventional MRI datasets.^7^

Structural disconnection can be estimated from subject-level lesion masks, easily derived from anatomical images, and population-averaged tractography atlases, without requiring individual diffusion imaging.^8^ Such atlas-based approaches have demonstrated substantial agreement with individual tractography-based disconnectomes,^9^ offering an alternative perspective for evaluating the effect of MS lesions. Disconnection metrics correlate with physical disability and systemic biomarkers of axonal damage,^10^ and disruption of specific brain subnetworks is linked to MS symptoms such as reduced information processing speed^11^ or depression.^12^

Similarly, single-subject GM networks can be built from anatomical MRI by estimating a set of morphological properties (e.g., volume, thickness, and curvature) within each cortical region and computing pairwise similarities.^13^ Different methods using this framework have demonstrated a restructuring of morphological similarity networks toward more disorganized configurations in pwMS, starting early in the disease,^14^ correlating with physical disability and cognitive impairment,^15^ and explaining disability worsening.^16^ However, these approaches have limitations, including how regions of interest are defined, the reliance on single metrics, or the reduction of complex data to simplistic summary statistics for each feature per region, making the link between GM networks and their neurobiological substrate somehow obscure.^17^ Recently, the Morphometric Inverse Divergence (MIND) method has been proposed that addresses these limitations by estimating within-subject similarity between cortical areas based on the divergence between their multivariate distributions of multiple MRI features, with the advantages of higher technical reliability and biological validity.^18^

Most studies have assessed structural disconnection and morphometric similarity in isolation, using small sample sizes or short follow-up periods. Consequently, their potential as biomarkers of MS severity and progression remains largely unexplored. Here, leveraging a large monocentric cohort of pwMS, we jointly mapped structural disconnection and morphometric similarity both cross-sectionally and longitudinally. We aimed to demonstrate whether the corresponding networks (1) are sensitive to MS-related brain damage and its progression over time; (2) can explain MS-related physical disability and cognitive dysfunction; and (3) can predict long-term clinical worsening.

## Methods

### Participants

In this longitudinal study, we retrospectively selected patients with a diagnosis of MS according to the 2010-McDonald criteria^19^ from the radiologic and clinical databases of the MS Center of the University of Naples “Federico II.” We included all pwMS who had undergone at least 1 structural brain MRI scan, comprising the same 3D-T1-weighted (T1w) sequence, between October 2006 and October 2020. In addition, we selected individuals who had participated as healthy controls (HC) in research studies during the same period and undergone the same MRI protocol. Exclusion criteria were age <18 or >75 years, and the presence of other neurologic, psychiatric, or systemic conditions.

### Clinical Evaluation

Clinico-demographic data of selected participants were retrieved from clinical and research records. For pwMS, physical disability and information processing speed were assessed within 1 week from the MRI using the Expanded Disability Status Scale (EDSS) and the Symbol Digit Modalities Test (SDMT), respectively. SDMT values were converted into age-, sex-, and education-adjusted *z*-scores based on normative values in the healthy population.^20^ For consistency with interpreting EDSS associations, SDMT *z*-scores were inverted before entering statistical analyses such that higher values reflected poorer cognitive performance. For patients who were followed up for more than 5 years, the latest available EDSS score was retrieved and confirmed disability progression (CDP) from the baseline examination was defined as an EDSS increase ≥1 (for baseline EDSS ≤5.5) or ≥0.5 (for baseline EDSS >5.5).^21^

### MRI Acquisition

All MRI scans were acquired on the same 3T scanner (Magnetom Trio; Siemens Healthineers, Erlangen, Germany), equipped with an 8-channel head coil. The acquisition protocol included a 3D T1w magnetization prepared rapid acquisition gradient echo sequence (repetition time [TR] = 1,900 milliseconds, echo time [TE] = 3.4 milliseconds, inversion time [TI] = 900 milliseconds, flip angle = 9°, voxel size = 1 × 1 × 1 mm^3^, 160 axial slices) for morphometric analyses and, for pwMS, a T2-weighted fluid-attenuated inversion recovery (T2w-FLAIR) sequence (3D: TR = 6,000 milliseconds, TE = 396 milliseconds, TI = 2,200 milliseconds, flip angle = 120°, voxel size = 1 × 1 × 1 mm^3^, 160 sagittal slices; or 2D: TR = 9,620 milliseconds, TE = 138 milliseconds, TI = 2,500 milliseconds, flip angle = 150°, voxel size = 1 × 1 × 3 mm^3^, 48 axial slices) for the assessment of demyelinating lesions.

### Lesion Segmentation and Structural Disconnection Networks

For all pwMS, demyelinating lesions were automatically segmented on T2w-FLAIR and T1w scans using the cross-sectional SAMSEG method in FreeSurfer v7.3.2.^22^ The obtained lesion masks were used to compute total lesion volume (TLV) and to fill lesions in T1w images for subsequent morphometric analyses through FSL's lesion-filling procedure.^23^ Individual lesion masks were registered to the MNI space by applying the nonlinear transformation obtained by normalizing T1w volumes to the template using ANTs v2.4.3.^24^ Spatially normalized lesion masks were used to obtain structural disconnection matrices based on a regional GM parcellation including 100 cortical regions from the Schaefer atlas^25^ and 14 subcortical regions FreeSurfer's segmentation.^26^ Using the Lesion Quantification Toolkit,^8^ which relies on the HCP-842 tractography atlas, the pairwise disconnection between structurally connected GM regions was computed as the proportion of streamlines intersecting lesions and used to fill subject-level 114 × 114 structural disconnection matrices. We generated group-level lesion and disconnection probability maps, expressing the probabilities of each voxel containing a lesion or at least 1 streamline intersecting a lesion, respectively.^8^ Nodes were assigned to 7 canonical functional system labels including visual (VIS), somatomotor (SM), dorsal attention (DAN), ventral attention (VAN), limbic, control (CONT), and default mode (DMN) networks,^27^ plus a network of subcortical regions. In addition, edges were aggregated into region-level features by summing all values attached to each node.

### Structural MRI Processing and Morphometric Similarity Networks

Lesion-filled T1w volumes were processed with FreeSurfer v6.0.1 using the recon-all cross-sectional pipeline implemented in the corresponding BIDS app.^26^ As for lesion segmentation, different MRI visits were considered as separate instances to apply identical image processing to all timepoints. Brain parenchymal fraction (BPF), considered a measure of global brain atrophy, was computed from FreeSurfer output as the ratio of brain volume to intracranial volume and expressed as *z*-scores adjusted for the effects of age and sex in healthy population. Based on FreeSurfer cortical surface reconstruction, vertex-level morphometric features (i.e., cortical thickness, GM volume, surface area, mean curvature, and sulcal depth) were extracted for 100 regions of interest defined by the Schaefer atlas^25^ and used to compute the pairwise morphometric similarity between cortical regions using the MIND approach.^18^ Briefly, MRI features were standardized across all vertices and aggregated to form regional multivariate distributions. The pairwise similarity between regional multivariate distributions was computed based on the symmetrized Kullback-Leibler divergence metric and bounded between 0 and 1, with higher values representing greater similarity. The obtained values were used to fill subject-level 100 × 100 cortical morphometric similarity matrices. As for structural disconnection matrices, aggregated network-level and region-level representations were also generated.

### Statistical Analysis

Unless otherwise specified, statistical analyses were performed using R (version 4.1.2). The effect of group (pwMS vs HC, only for morphometric similarity networks), EDSS, and SDMT scores on baseline structural disconnection and morphometric similarity networks were tested with the network-based statistics (NBS) approach, as implemented in the NBR package.^28^ NBS is a nonparametric method for performing statistical analysis on networks, that adjusts for multiple comparisons by clustering within the topological (rather than physical) space. Briefly, (1) the hypothesis of interest is tested edge-wise using the general linear model; (2) connections are filtered according to a test statistic threshold; (3) connected graph components are identified among suprathreshold connections; and (4) a family-wise error (FWE)–corrected *p*-value is computed for each component based on the sum of test statistic values using permutation testing.^28^ Similarly, longitudinal changes of structural disconnection and morphometric similarity networks were assessed using the implementation of linear mixed-effects models for NBS provided by the NBR package, with time points nested within participants and random intercept and slope of follow-up time per subject. Similar mixed-effects models were used to assess the longitudinal evolutions of EDSS, TLV (log(x + 1)-transformed to account for the positively skewed distribution), and *z*-scored BPF. For all NBS analyses, baseline age, age^2^ (to account for the nonlinear effect of age), and sex were included in the model as nuisance variables, with a primary statistical threshold of *p* < 0.01, 5,000 permutations, and a statistical significance level set at *p*_FWE_ < 0.05. As we were interested in subnetwork-specific effects rather than the influence of global lesion burden or atrophy, models assessing the correlations with clinical variables were additionally adjusted for log(x + 1)-transformed TLV (for structural disconnection matrices) and BPF *z*-scores (for morphometric similarity matrices).To confirm that we were effectively filtering out global effects driven by overall lesion burden and atrophy, we also repeated these analyses without adjustments. When subnetworks exhibiting significant change over time emerged, these were summarized for further analyses by *z*-scoring each edge using the healthy population as a reference and calculating the average of their modules to obtain global synthetic measures of longitudinal structural disconnection and morphometric similarity alteration. We used absolute *z*-score values because we were interested in quantifying overall deviation from the healthy norm, precluding effects of opposite sign from canceling each other out. We used Spearman rank correlation to test the associations of the identified subnetworks with annualized EDSS change. In addition, the associations between altered subnetworks and disability worsening were reassessed while accounting for changes in log(x + 1)-transformed TLV and BPF *z*-scores, respectively, using partial correlations.

To evaluate the prognostic value of structural disconnection and morphometric similarity, we tested whether baseline networks could predict long-term CDP using the NBS-predict approach, a prediction-based extension of NBS combining machine learning models with connected components in a cross-validation (CV) structure, as implemented in the corresponding MATLAB (MathWorks, 2017) toolbox.^29^ We ran NBS-predict with 5-fold nested CV (primary threshold *p* < 0.01) and hyperparameter optimization using Bayesian optimization with 100 iterations. For a complete list of hyperparameters and explored ranges, we refer the reader to the NBS-predict manual.^30^ The CV structure was repeated 10 times to reduce the variation in the model performance estimation. We scaled data and regressed out baseline age, age^2^, sex, and log-transformed TLV (for structural disconnection matrices) and BPF *z*-scores (for morphometric similarity matrices), using a cross-validated deconfounding technique to prevent data leakage.^29^ Different machine learning algorithms (logistic regression, linear support vector classification, and linear discriminant analysis) were evaluated, with classification accuracy as the performance metric and 500 permutations to assess the significance of the models' predictions.^29^

Finally, we investigated the coupling between structural disconnection and morphometric similarity in pwMS at baseline at different scales using Spearman rank correlation. At the global network level, we determined the group-level correlation between mean structural disconnection and mean morphometric similarity across participants. At the edge level, we averaged structural disconnection and morphometric similarity networks across participants and correlated the 2 vectorized matrices. At the node level, regional connectivity profiles were extracted row-wise from structural disconnection and morphometric similarity matrices and correlated with each other to obtain coupling values for each of the 100 cortical parcels. To investigate the possible effect of disease phase, we split the patients based on the median disease duration (DD) and assessed network coupling separately in pwMS with shorter vs longer DD.

### Standard Protocol Approvals, Registrations, and Patient Consents

The study was conducted in compliance with the Declaration of Helsinki and approved by the Ethics Committee “Carlo Romano” of the Host Institution. Written informed consent was obtained from all participants.

### Data Availability

Derived data that support the findings of this study are available from the corresponding author on reasonable request. Raw data are not available because of reasons of sensitivity.

## Results

### Participants

We analyzed 461 pwMS (F/M = 324/137, mean age at baseline = 37.2 ± 10.6 years, mean DD at baseline = 9.1 ± 7.9 years, clinical phenotype at baseline = 387 relapsing-remitting, 51 secondary-progressive, 23 primary-progressive) corresponding to 1,235 visits (median number of visits per patient = 4, range = 1–8; mean follow-up time = 1.9 ± 2.0 years, range = 0.1–13.3 years), and 55 HCs (F/M = 25/30, mean age = 42.4 ± 15.7 years).

Baseline EDSS (median = 2.5, interquartile range = 2.0–4.0) and SDMT (mean *z*-score = −1.1 ± 1.1) were available for 459 and 247 pwMS, respectively. Long-term clinical follow-up was available for 285 pwMS (mean follow-up time = 12.4 ± 2.8 years, range = 5.1–17.2 years) (eFigure 1), 127 of whom (44.6%) exhibited CDP. Demographic, clinical, and MRI characteristics of the studied population are reported in the Table.

**Table:** Baseline Demographic and Clinical Characteristics of the Studied Population

	Healthy controls (N = 55)	Patients with multiple sclerosis (N = 461)	*p* Value
Age, y	42.4 (15.7)	37.2 (10.6)	0.02
Sex, F/M	25/30	324/137	<0.001
Disease duration, y	—	9.1 (7.9)	n.a.
Clinical phenotype, relapsing-remitting/secondary-progressive/primary-progressive	—	387/51/23 (84/11/5)	n.a.
Disease-modifying therapy, first-line/second-line/no therapy	—	257/110/94 (56/24/20)	n.a.
Expanded Disability Status Scale	—	2.5 (2.0–4.0)^a^	n.a.
Symbol Digit Modalities Test, *z*-score	—	−1.1 (1.1)^b^	n.a.
Total lesion volume, mm^3^	—	7,997.9 (8,832.8)	n.a.
Brain parenchymal fraction, *z*-score	0.0 (1.0)	−2.1 (2.7)	<0.001

Data are expressed as mean (SD), except for Expanded Disability Status Scale which is expressed as median (interquartile range). Between-group differences were tested with either Welch *t* test (age and brain parenchymal fraction) or χ^2^ (sex) tests.

a N = 459.

b N = 247.

### Structural Disconnection and Morphometric Similarity Networks in Patients With MS Compared With HC

**Figure 1:**
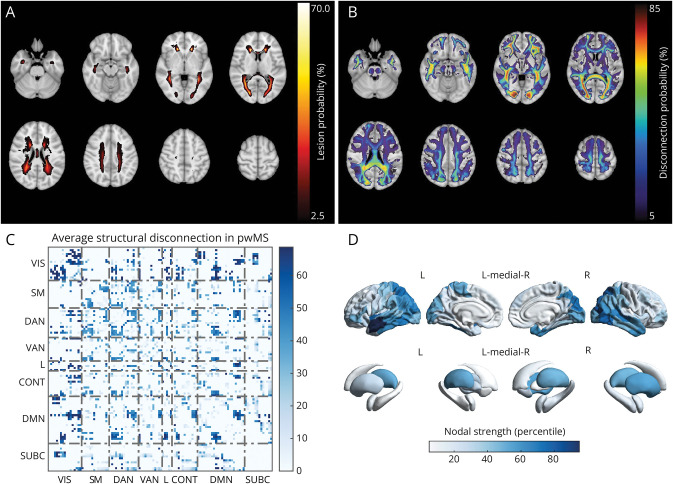
Structural Disconnection in pwMS Group-level lesion (A) and disconnection (B) probability maps in pwMS, expressing the probabilities of each voxel containing a lesion or at least 1 streamline intersecting a lesion, respectively. Network-level (C) and region-level (D) representations of average structural disconnection in pwMS. CONT = control network; DAN = dorsal attention network; DMN = default mode network; L = limbic network; pwMS = patients with multiple sclerosis; SM = somatomotor network; SUBC = subcortical network; VAN = ventral attention network; VIS = visual network.

At baseline, pwMS displayed the highest lesion probability in the periventricular WM (Figure 1A), with the highest disconnection probability at the level of the occipital WM, splenial commissural fibers, and long-range frontal and temporal association tracts (Figure 1B). On average, structural disconnection was mainly observed between the VIS and the SM and nonsensorimotor networks, as well as within and between cortical associative networks and around subcortical structures (Figure 1C). At the regional level, the most structurally disconnected nodes were the thalami and temporal and posterior cortical regions (Figure 1D).

**Figure 2:**
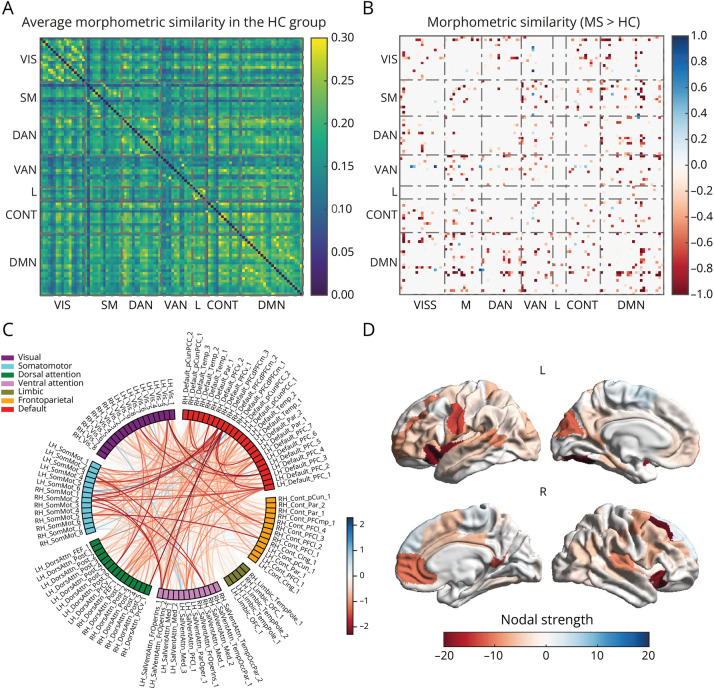
Morphometric Similarity Disruption in pwMS Average morphometric similarity network in the HC group (A). Network-level (B), edge-level (C), and region-level (D) representations of the subnetwork of significant between-group differences in morphometric similarity (pwMS > HC). CONT = control network; DAN = dorsal attention network; DMN = default mode network; HC = healthy controls; L = limbic network; pwMS = patients with multiple sclerosis; SM = somatomotor network; VAN = ventral attention network; VIS = visual network.

Compared with HC (Figure 2A), pwMS showed a distributed subnetwork of predominantly disrupted morphometric similarity (431 edges, *p*_FWE_ < 0.01, Figure 2, B and C), with the prominent involvement of occipital, pericentral, perisylvian, and prefrontal cortices (Figure 2D). Global structural disconnection and morphometric similarity disruption significantly correlated with TLV (Spearman ρ = 0.94, *p* < 0.001, eFigure 1A) and BPF (Spearman ρ = −0.40, *p* < 0.001, eFigure 1B), respectively.

### Baseline Associations of Structural Disconnection and Morphometric Similarity Networks With Physical and Cognitive Disability

**Figure 3:**
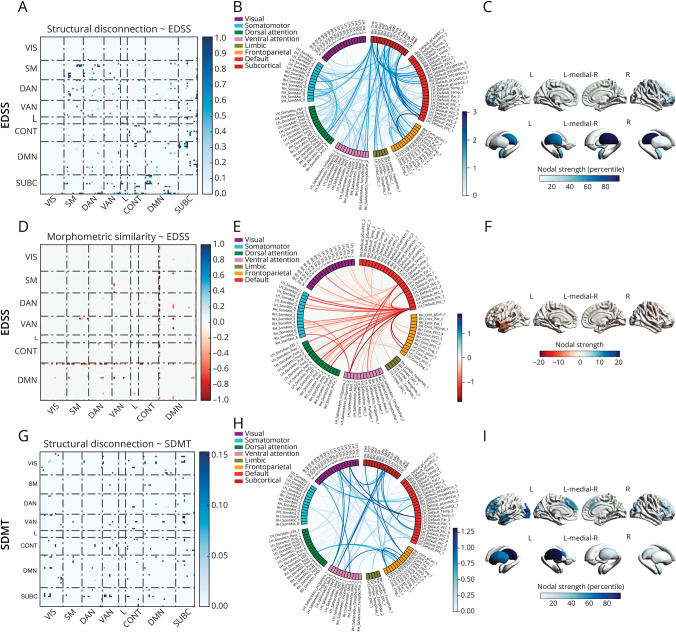
Structural Disconnection and Morphometric Similarity Disruption Explain Physical and Cognitive Disability Network-level (A), edge-level (B), and region-level (C) representations of the subnetwork of significant association between EDSS and structural disconnection. Network-level (D), edge-level (E), and region-level (F) representations of the subnetwork of significant association between EDSS and morphometric similarity. Network-level (G), edge-level (H), and region-level (I) representations of the subnetwork of significant association between SDMT and structural disconnection. CONT = control network; DAN = dorsal attention network; DMN = default mode network; EDSS = expanded disability status scale; L = limbic network; SDMT = Symbol Digit Modalities Test; SM = somatomotor network; SUBC = subcortical network; VAN = ventral attention network; VIS = visual network.

At baseline, we found a subnetwork of significant association between EDSS and structural disconnection (225 edges, *p*_FWE_ < 0.01), mainly involving cortico-subcortical tracts, within-transmodal connections of the DMN, the DAN, the VAN and the CONT, and links between these and sensorimotor networks. The regions participating the most in this subnetwork were the thalami, the amygdalae, and the prefrontal cortex (Figure 3, A–C). EDSS was also significantly associated with a smaller subnetwork of predominantly disrupted morphometric similarity between the DMN and the other networks, with the prominent participation of the insula and the perisylvian cortex of the left hemisphere (86 edges, *p*_FWE_ = 0.02) (Figure 3, D–F). Similarly, SDMT was associated with a relatively small subnetwork of predominantly cortico-subcortical structural disconnection mostly involving the left hemisphere (88 edges, *p*_FWE_ < 0.01), with the participation of the thalamus and prefrontal, temporal, and occipital cortical regions (Figure 3, G–I). No significant subnetworks emerged when assessing the relationship between morphometric similarity and SDMT. When repeating the analyses without adjusting for global lesion burden and brain atrophy, we observed more distributed, less circuit-specific, effects (eAppendix 1 and eFigure 2).

### Longitudinal Changes of Structural Disconnection and Morphometric Similarity and Association With Disability Worsening

**Figure 4:**
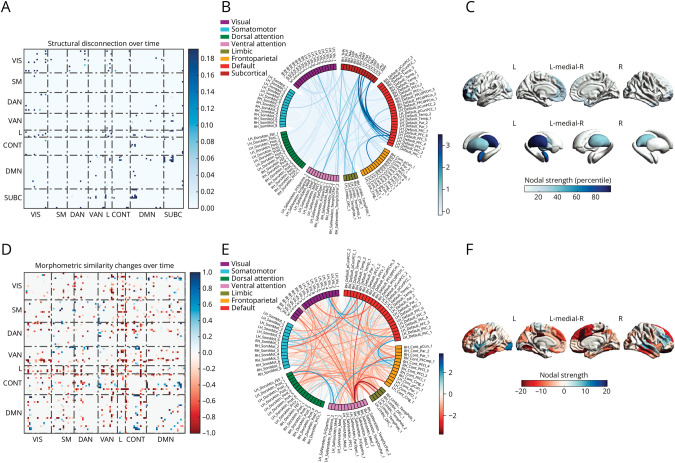
Structural Disconnection and Morphometric Similarity Changes Over Time Network-level (A), edge-level (B), and region-level (C) representations of the subnetwork of significant structural disconnection over time in pwMS. Network-level (D), edge-level (E), and region-level (F) representations of the subnetwork of significant morphometric similarity change over time in pwMS. CONT = control network; DAN = dorsal attention network; DMN = default mode network; L = limbic network; pwMS = patients with multiple sclerosis; SM = somatomotor network; SUBC = subcortical network; VAN = ventral attention network; VIS = visual network.

Longitudinally, we found a smaller subnetwork of progressive structural disconnection (82 edges, *p*_FWE_ = 0.04) involving mainly fronto-thalamic tracts (Figure 4, A–C). Moreover, we observed a larger and anatomically distributed subnetwork of progressive morphometric similarity alterations (509 edges, *p*_FWE_ < 0.01), comprising pairs of regions exhibiting both increased and decreased similarity over time (Figure 4, D–F). Longitudinal models also showed significant EDSS worsening (B = 0.084, SE B = 0.015, *p* < 0.01), and whole-brain volume loss (B = −0.095, SE B = 0.022, *p* < 0.01) over time, while the increase in global lesion burden was not significant (B = 0.003, SE B = 0.004, *p* = 0.50). Annualized structural disconnection significantly correlated with annualized changes in EDSS scores (Spearman ρ = 0.07, *p* = 0.02), with this correlation remaining significant after adjusting for longitudinal TLV change (Spearman ρ = 0.08, *p* = 0.004). Similarly, individualized morphometric similarity change per year correlated with annualized EDSS change (Spearman ρ = 0.11, *p* < 0.001), with this correlation remaining significant also after accounting for annualized BPF change (Spearman ρ = 0.09, *p* = 0.002).

### Baseline Structural Disconnection and Morphometric Similarity Networks and Long-term Disability Progression

**Figure 5:**
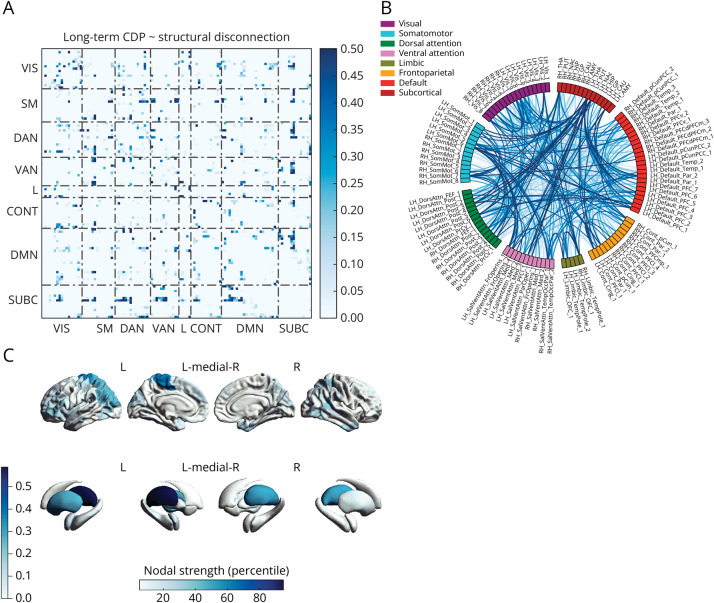
Baseline Structural Disconnection Predicts Long-Term Disability Progression Network-level (A), edge-level (B), and region-level (C) representations of the structural disconnection subnetwork predicting long-term CDP. CDP = confirmed disability progression. CONT = control network; DAN = dorsal attention network; DMN = default mode network; L = limbic network; SM = somatomotor network; SUBC = subcortical network; VAN = ventral attention network; VIS = visual network.

Using NBS-predict with the baseline structural disconnection matrices as input, a linear support vector machine classifier significantly predicted long-term CDP (710 edges, accuracy = 0.59, 95% CI = 0.58–0.60, *p* = 0.03), with modest sensitivity (0.49) but good specificity (0.67). The identified subnetwork mainly involved cortico-subcortical tracts, within-transmodal connections of the DMN, the DAN, the VAN, and the CONT, and links between these and sensorimotor networks. The regions participating the most in this subnetwork were preferentially located in the left hemisphere and included the thalamus and the parieto-occipital, pericentral, and prefrontal cortices (Figure 5). Models relying on baseline morphometric similarity matrices did not achieve above chance-level accuracy for the prediction of CDP.

### Coupling Between Structural Disconnection and Morphometric Similarity

**Figure 6:**
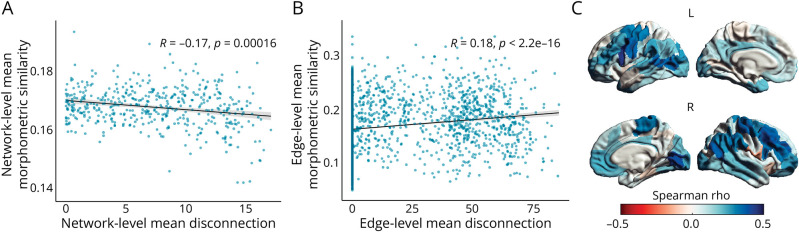
Coupling Between Structural Disconnection and Morphometric Similarity Network-level (A), edge-level (B), and region-level (C) associations between structural disconnection and morphometric similarity. (A) Group-level correlation between mean structural disconnection and mean morphometric similarity across participants. (B) Edge-level correlation between average structural disconnection and morphometric similarity networks. (C) For each of the 100 cortical parcels, correlation between regional structural disconnection and morphometric similarity profiles.

At the global network level, higher mean structural disconnection in pwMS was associated with lower mean morphometric similarity (Spearman ρ = −0.18, *p* < 0.001) (Figure 6A). Conversely, at a more granular level, edges with a greater probability of structural disconnection were generally associated with higher morphometric similarity (Spearman ρ = 0.18, *p* < 0.001), with the distribution of values suggesting a nonlinear, multiphasic, relationship between the 2 (Figure 6B and eFigure 3). Similarly, at the node level, there was a positive association between regional structural disconnection and morphometric similarity profiles (average Spearman ρ = 0.16, *p* < 0.001) with the strongest coupling observed at the level of sensorimotor areas and fronto-parietal association hubs (Figure 6C). As these associations were seemingly driven by nondisconnected regions (showing, on average, lower morphometric similarity), we reassessed regional coupling after excluding links with no disconnection. This post hoc analysis revealed a slight negative edge-level coupling (Spearman ρ = −0.084, *p* < 0.01), with associations in both directions at the node level (eFigure 4). When investigating the possible effect of disease phase, at the global level, a negative relationship between mean structural disconnection and morphometric similarity only emerged in pwMS with longer DD (Spearman ρ = −0.36, *p* < 0.001), likely driven by the greater lesion load and whole-brain atrophy, with no significant association in the shorter DD group (Spearman ρ = 0.076, *p* = 0.25). Conversely, at the regional levels, the direction and magnitude of the coupling were very similar across groups (eAppendix 1 and eFigure 5).

## Discussion

Using network analysis methods and conventional MRI sequences, we identified patterns of structural disconnection and morphometric similarity disruption in the brains of pwMS. These measures proved to be sensitive to disease progression, explained clinical disability, and predicted its long-term evolution, independently from global lesion burden and atrophy.

In line with well-established evidence,^31^ the highest T2 lesion occurrence in our cohort was observed in the periventricular WM, probably reflecting the preferential perivenular distribution of MS-related inflammatory demyelination. As the anatomical configurations of long-range and cortico-subcortical tracts make them more likely to traverse lesional areas, structural disconnection was mainly centered around the thalami and cortical sensorimotor (occipital and pericentral cortices) and associative (temporal cortex) hubs. The thalamus, in particular, with its multiple reciprocal connections, is sensitive to lesions occurring in multiple regions, thus acting as a “barometer” for diffuse parenchymal damage.^32^ Similarly, the visual and sensorimotor cortices, as well as the temporal cortex, are served by long-range tracts sustaining their function as primary sensory areas and integration hubs, respectively, thus being particularly prone to structural disconnection.^33^

While TLV and average structural disconnection are linked by a logarithmic relationship enforced by the brain's geometry, not all lesion locations bear equal clinical relevance, differentially affecting physical disability,^34^ cognition,^35^ and long-term clinical outcomes.^36^ The examination of network-level effects, using structural disconnectomes, adds information on the effect of MS lesions, potentially resulting in more robust neurobiological and clinical associations compared with the assessment of isolated regions/connections.^37^ Indeed, we found that, independent of the global lesion burden, physical disability and cognition were explained by disconnection within specific subnetworks. In particular, higher EDSS was associated with greater structural disconnection centered around the thalami and frontal cortices, confirming previously reported relations between physical disability and disruption of fronto-thalamic and frontal commissural pathways.^34,38,39^ Similarly, worse performances at the SDMT were mainly explained by structural disconnection involving fronto-thalamic and frontal commissural tracts, but also long-range occipito-frontal and temporo-frontal association tracts, mostly in the left hemisphere. The relevance of thalamo-cortical, commissural, and long-range association tracts for cognitive functioning has been highlighted with various approaches looking at lesion location or WM microstructural properties.^33,35^ Our results confirm that atlas-based lesion disconnectomics is sensitive to these effects, providing additional information compared with lesions and regional properties' assessments.^11,40^

Morphometric similarity networks were also sensitive to MS-related brain damage, with an anatomically distributed subnetwork of similarity disruption in pwMS at baseline. The brain's intrinsic structural organizing principles result in remote regions sharing macroscale morphological traits, thereby establishing a network of morphological similarity that can be imaged using structural MRI.^13^ Using the MIND approach, we showed that a relatively disordered phenomenon such as MS-related neurodegeneration can disrupt this genetically determined organization, resulting in an overall reduction of morphological similarity. These results confirm previous evidence of a more random organization of single-subject GM networks in pwMS,^14,15^ with the advantage of a method that measures multiple morphological properties simultaneously with vertex-level details and natively aligns with macroscale brain parcellations, therefore being more neurobiologically grounded. Morphometric similarity disruption centered around the left perisylvian cortex explained physical disability beyond whole-brain atrophy, which might speculatively be interpreted considering the reported complex and clinically relevant anatomo-functional alterations of attentional and default networks in MS.^4,41^

Longitudinally, we identified a subnetwork of progressive structural disconnection comprising fronto-thalamic tracts, explaining disability worsening independently from lesion accrual. These results further highlight the role of cortico-thalamic connections in MS,^39^ confirming that assessing network-level structural disconnection yields clinically relevant information beyond mere lesion burden. Similarly, morphometric similarity networks were sensitive to longitudinal changes in the brain's structure. Along with edges of longitudinally decreasing morphological similarity, we found pairs of regions whose morphological traits tended to match over time, substantially aligning with previously described atrophy patterns encompassing the middle temporal gyrus and sensorimotor cortices,^42^ as well as insular and prefrontal cortices and the occipital pole.^43^ Indeed, despite being a relatively disordered phenomenon, MS-related neurodegeneration is not completely random, with different spatial patterns of atrophy described in association with MS.^42,43^ This disease-related structural covariance, likely constrained by network-based mechanisms and shared vulnerability,^44^ may explain the observed increase in morphometric similarity. Of interest, the longitudinal changes in morphometric similarity paralleled disability worsening independently from whole-brain volume loss, confirming its ability to capture additional information.

We also evaluated the prognostic value of the assessed networks. Previous attempts have been made to predict individual-level prognosis using lesion location,^36^ or regional network measures.^11,16^ Using NBS-predict, we demonstrated that baseline individual structural disconnectomes can be used to significantly predict long-term disability progression independently from global lesion burden. Although the model's predictive performance did not meet clinical acceptability standards, it was shown that the disconnection of distinct subnetworks may serve as a specific marker of clinical progression. Conversely, the observed low sensitivity may be attributed to the correction for global lesion burden (filtering only subnetwork-specific effects) and the protracted follow-up period, during which numerous unaccounted-for superadded factors (including disconnection) might have emerged. Structural disconnections of the thalamus and the paracentral lobule were the most important predictors of disability worsening, with a slight predilection for the left hemisphere. While this confirms the major role of thalamic disconnection in sustaining physical disability,^38^ the association between long-term disability progression and structural disconnection of the paracentral lobule, including the primary sensorimotor areas of the lower limbs, may be related to the known heavy dependence of EDSS on motor function and walking ability.^45^ On the other hand, long-term disability progression was not predictable from baseline morphometric similarity networks, possibly because they did not account for deep GM damage, which is known to bear great prognostic relevance.^46^

Finally, we investigated the relationship between the 2 explored network domains. At the global level, where mean structural disconnection and morphometric similarity primarily reflect the overall severity of brain damage, greater structural disconnection was associated with more disrupted morphometric similarity. This relationship only emerged in more advanced phases of the disease, likely driven by the more severe global lesion burden and brain atrophy. Conversely, at more granular levels, structural disconnection and morphometric similarity exhibited a complex relationship. While nondisconnected regions were, on average, less morphologically similar than other node pairs, progressive structural disconnection appeared to be associated with a slight decrease in morphometric similarity. These results may indicate a nonmonophasic relationship between the 2 domains. Initially, disconnected nodes may exhibit similar morphological changes, with structural disconnection potentially shaping patterns of concerted neurodegeneration across GM regions through lesion-related transneuronal degeneration inducing similar atrophic changes at both ends of the disrupted WM tract.^44^ Subsequently, more pronounced disconnection may disrupt this morphological covariance and result in diminished morphometric similarity. Nevertheless, it is important to note that at least a portion of the nondisconnected node pairs may not be anatomically connected (i.e., they may have no connecting fibers to be potentially disrupted), and therefore intrinsically less morphologically similar.

Our study has some limitations. While the atlas-based assessment of structural disconnection has been validated and has the advantage of greater accessibility, diffusion MRI-based tractography remains the gold standard and would have further strengthened our results. In addition, the MIND approach in its standard formulation does not allow the inclusion of subcortical GM, which is known to be highly relevant in MS, prompting the development of methodological advances to meaningfully incorporate deep GM structures in morphological similarity analyses. Assessing structural disconnection and morphometric similarity changes in relation to finer clinical outcomes would help identify domain-specific network alterations, potentially informing treatment targeting. Moreover, additional research relying on advanced statistical methods and prospective designs is needed to establish any causal relationships between the reported changes and to model their patterns of progression throughout the disease course. Finally, given the lack of longitudinal data for the HC, we cannot fully exclude that the observed longitudinal changes in pwMS were, at least partially, due to physiologic processes (i.e., aging). However, it has been shown that while the adolescent brain undergoes relevant longitudinal changes,^47^ there seem to be less substantial morphometric similarity changes in the adult brain, especially in the age range of our sample, with only minor deviations even in psychiatric populations.^48^ While this evidence cannot be fully generalized to our sample, it seems reasonable to speculate that the observed longitudinal changes were mostly driven by disease-related processes. Nonetheless, further studies are needed to disentangle age-related from disease-specific morphometric similarity longitudinal changes.

In conclusion, our results show that networks of structural disconnection and morphometric similarity obtained from conventional MRI are sensitive to MS-related brain damage and its progression over time, potentially providing complementary information to other established MRI-derived biomarkers of disease severity and progression. Extracting network measures from conventional MRI scans holds the potential for bridging the gap between connectomics and clinical practice, driving advanced network analyses toward real-world applicability.

## Glossary

BPF: brain parenchymal fraction
CDP: confirmed disability progression
CONT: control network
CV: cross-validation
DAN: dorsal attention network
DD: disease duration
DMN: default mode network
EDSS: Expanded Disability Status Scale
FLAIR: fluid-attenuated inversion recovery
FWE: family-wise error
GM: gray matter
HC: healthy control
MIND: morphometric inverse divergence
MPRAGE: magnetization-prepared rapid acquisition gradient echo sequence
MS: multiple sclerosis
NBS: network-based statistics
pwMS: people with MS
SDMT: Symbol Digit Modalities Test
SM: somatomotor network
T1w: T1-weighted
T2w: T2-weighted
TE: echo time
TI: inversion time
TLV: total lesion volume
TR: repetition time
VAN: ventral attention network
VIS: visual
WM: white matter
